# Sex-specific difference of in-hospital mortality from COVID-19 in South Korea

**DOI:** 10.1371/journal.pone.0262861

**Published:** 2022-01-24

**Authors:** Ae-Young Her, Youngjune Bhak, Eun Jung Jun, Song Lin Yuan, Scot Garg, Semin Lee, Jong Bhak, Eun-Seok Shin

**Affiliations:** 1 Department of Internal Medicine, Kangwon National University School of Medicine, Chuncheon, South Korea; 2 Department of Biomedical Engineering, College of Information-Bio Convergence Engineering, Ulsan National Institute of Science and Technology (UNIST), Ulsan, South Korea; 3 Department of Internal Medicine, Ulsan University Hospital, University of Ulsan College of Medicine, Ulsan, South Korea; 4 Department of Cardiology, Dong-A University Hospital, Busan, South Korea; 5 East Lancashire Hospitals NHS Trust, Blackburn, Lancashire, United Kingdom; Kurume University School of Medicine, JAPAN

## Abstract

We sought to assess the impact of sex on in-hospital mortality of patients with COVID-19 infection in South Korea. The study recruited 5,628 prospective consecutive patients who were hospitalized in South Korea with COVID-19 infection, and enrolled in the Korea Centers for Disease Control and Prevention (KCDC) dataset between January 20, 2020, and April 30, 2020. The primary endpoint was in-hospital death from COVID-19. The cohort comprised of 3,308 women (59%) and 2,320 men (41%). In-hospital death was significantly lower in women than men (3.5% vs. 5.5%, hazard ratio (HR): 0.61; 95% confidence interval (CI): 0.47 to 0.79, p <0.001). Results were consistent after multivariable regression (HR: 0.59; 95% CI: 0.41 to 0.85, p = 0.023) and propensity score matching (HR: 0.51; 95% CI: 0.30 to 0.86, p = 0.012). In South Korea, women had a significantly lower risk of in-hospital death amongst those patients hospitalized with COVID-19 infection.

## Introduction

Since the outbreak of coronavirus disease 2019 (COVID-19) in Wuhan, China in December 2019, it has rapidly spread around the world [1–3]. As of June 11, 2021, the World Health Organisation (WHO) reported a total of 174,789,927 COVID-19 cases globally, with an average mortality of 2.2%. Experience from past outbreaks highlights the importance of incorporating a sex analysis into the preparedness and response efforts to improve the effectiveness of health interventions, and promote sex and health equity goals [4]. Interestingly, a recent analysis of COVID-19 data from 188 countries has shown sex-specific mortality differences with higher death rates in men compared with women, although the underlying mechanisms are unclear [5]. However, these findings are not consistent in all countries, and sex-specific morbidity and mortality vary among different countries. This would imply that there are geographic, genetic, cultural and/or sex-specific differences that may influence the spread of COVID-19 and subsequent mortality.

Therefore, the aim of this study was to assess the impact of sex on in-hospital mortality amongst patients hospitalized with COVID-19 infection in South Korea.

## Materials and methods

### Study population and endpoint

This prospective consecutive cohort study using the Korea Centers for Disease Control and Prevention (KCDC) dataset enrolled patients with a history of hospitalization for COVID-19 between January 20, 2020, and April 30, 2020, in South Korea. All patients with COVID-19 were diagnosed and treated according to the guidelines published by the KCDC (http://www.cdc.go.kr) [6]. The reverse transcription polymerase chain reaction test was used for detecting COVID-19, and nasal and pharyngeal swab specimens were collected from patients. A person who tested positive was confirmed to be infected with COVID-19 regardless of the presence of any clinical symptoms. The primary endpoint was in-hospital death during the hospital stay. All the patients analyzed either died in hospital or were discharged home despite the limitations that we could not acquire the information for in-hospital mortality due to other causes except COVID-19 or underlying diseases [6].

### Statistical analysis

An extended description of the statistical analysis is presented in the Online Appendix. Cumulative event rates were calculated based on Kaplan-Meier censoring estimates, and comparison of clinical outcomes between women and men was performed with the log-rank test. Because differences in baseline characteristics could significantly affect outcomes, sensitivity analyses were performed to adjust for confounders as much as possible. First, a multivariable Cox regression model was used. Covariates in the multivariable model were selected if they were significantly different between women and men or had predictive values. Cox proportional hazard models for primary end point satisfied the proportional hazards assumption. Second, the logistic regression method was used in a propensity score matching. Propensity score matching yielded 621 patients in women and 621 control subjects in men. Balance between the 2 groups after propensity score matching was assessed by calculating standardized mean differences. The standardized mean differences after propensity score matching were within 0.1 across all matched covariates, demonstrating successful balance achievement between comparative groups (S1 Table in S1 File). To identify independent predictors of in-hospital death, we used a multivariable Cox proportional hazard model. C-statistics with 95% confidence intervals (CIs) were calculated to validate the discriminant function of the model. In addition, comparisons of the primary end point between women and men according to the exploratory subgroups of interest were followed, and the interaction between treatment effect and these covariates was assessed with a Cox regression model. Statistical analyses were performed using R version 4.0.2 (R Foundation for Statistical Computing, Vienna, Austria). All probability values were 2-sided, and p value <0.05 was considered statistically significant. The data can be accessed for analysis once approved by Korea Centers for Disease Control and Prevention (https://is.kdca.go.kr/).

### Ethical approval

This study was approved by the ethics committee of the) KCDC and written informed consent was exempted because of the de-identified retrospective nature of the publicly available data.

## Results

**Fig 1:**
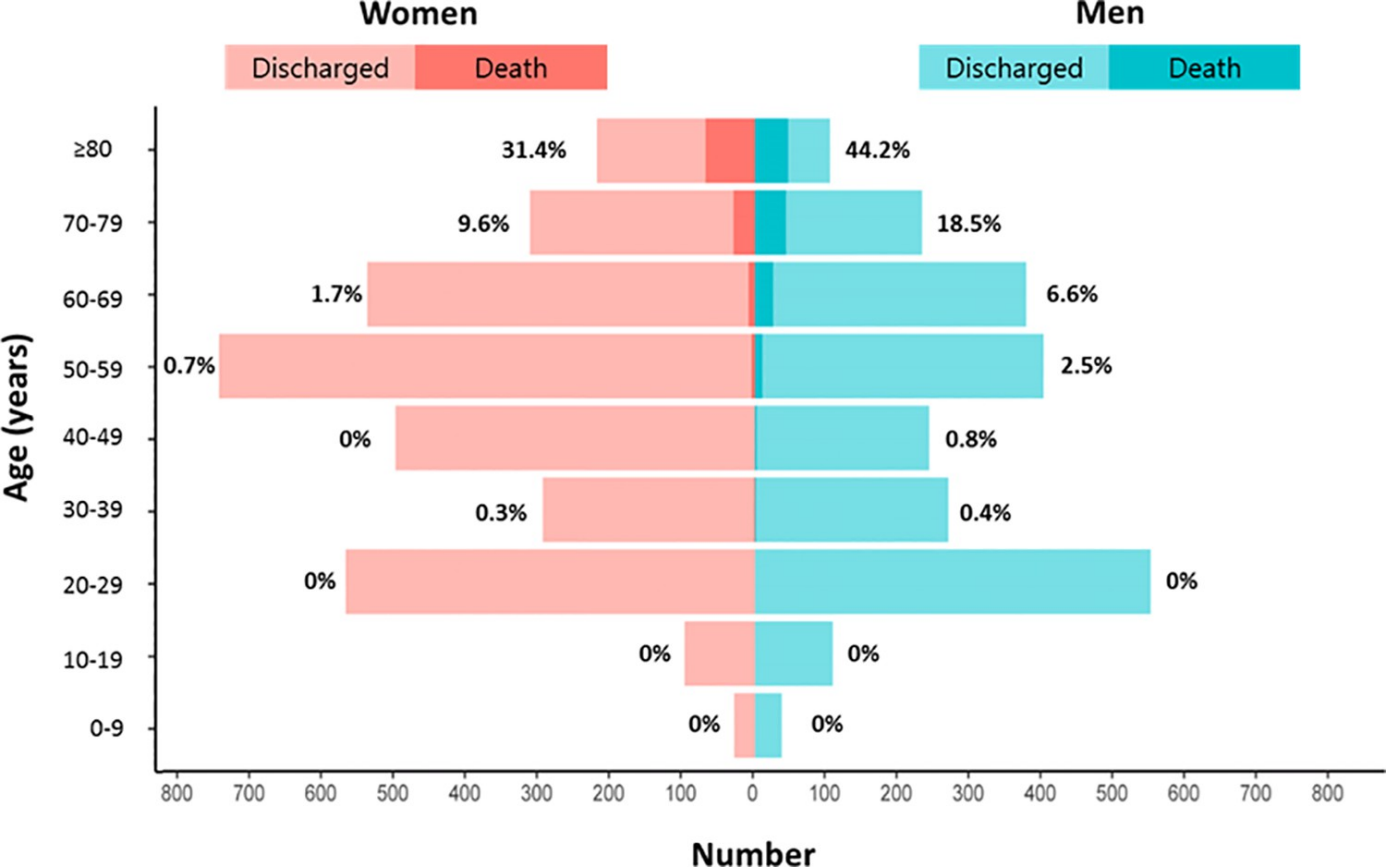
The prevalence of COVID-19 infection and in-hospital mortality according to sex and age. Data are percentage of in-hospital death. The bright and dark red boxes represent the number of discharged and in-hospital deaths in women. The bright and dark blue boxes represent the number of discharged and in-hospital deaths in men.

Baseline clinical characteristics of the patients are shown in Table 1. A total of 5,628 patients with confirmed COVID-19 infection were included in the KCDC dataset during the pandemic period. Confirmed cases were more frequent in women (n = 3,308, 59%) than in men (n = 2,320, 41%). The age distribution between the sexes was significantly different (Table 1 and Fig 1). The proportion of middle-aged (40 to 59 years old; 37.6% vs. 27.7%, p <0.001) and very-elderly (>80 years old) patients (6.7% vs. 4.5%, p <0.001) was higher in women compared with men. The percentage of underweight (6.7% vs. 4.7%, p = 0.004) and overweight (18.6% vs. 30.9%, p <0.001) patients was significantly higher in women and men, respectively. The prevalences of systolic blood pressure (SBP) ≥130 mmHg (49.6% vs. 63.0%, p <0.001) and diastolic blood pressure (DBP) ≥80 mmHg (58.7% vs. 65.6%, p <0.001) were significantly higher in men. The prevalence of diabetes mellitus (DM) (11.1% vs. 14.0%, p <0.001), chronic obstructive pulmonary disease (COPD) (0.5% vs. 1.1%, p = 0.006), and chronic liver disease (1.1% vs. 2.2%, p = 0.002) were lower in women compared with men. Dementia was more frequent (4.9% vs. 3.2%, p = 0.002) in women compared with men.

**Table 1:** Baseline clinical characteristics and outcomes.

Variables	Total (n = 5,628)	Women (n = 3,308)	Men (n = 2,320)	p-value
**Age, years**				<0.001
** 0–9**	66 (1.2)	29 (0.9)	37 (1.6)	0.014
** 10–19**	206 (3.7)	98 (3.0)	108 (4.7)	<0.001
** 20–29**	1119 (19.9)	569 (17.2)	550 (23.7)	<0.001
** 30–39**	564 (10.0)	295 (8.9)	269 (11.6)	<0.001
** 40–49**	742 (13.2)	500 (15.1)	242 (10.4)	<0.001
** 50–59**	1146 (20.4)	745 (22.5)	401 (17.3)	<0.001
** 60–69**	916 (16.3)	539 (16.3)	377 (16.2)	0.965
** 70–79**	545 (9.7)	313 (9.5)	232 (10.0)	0.502
** ≥80**	324 (5.8)	220 (6.7)	104 (4.5)	<0.001
**BMI, kg/m** ^**2**^				<0.001
** <18.5 (underweight)**	260 (5.9)	173 (6.7)	87 (4.7)	0.004
** 18.5–24.9 (normal)**	2,906 (65.7)	1,805 (70.4)	1,101 (59.1)	<0.001
** 25.0–29.9 (overweight)**	1,052 (23.8)	476 (18.6)	576 (30.9)	<0.001
** ≥30.0 (obesity)**	208 (4.7)	109 (4.3)	99 (5.3)	0.100
**SBP, mmHg**				<0.001
** <130**	2,462 (44.9)	1,629 (50.4)	833 (37.0)	<0.001
** ≥130**	3,024 (55.1)	1,603 (49.6)	1,421 (63.0)	<0.001
**DBP, mmHg**				<0.001
** <80**	2,113 (38.5)	1,335 (41.3)	778 (34.5)	<0.001
** ≥80**	3,373 (61.5)	1,897 (58.7)	1,476 (65.5)	<0.001
**Heart rate, beats/min**	86 ± 15	85 ± 15	86 ± 15	0.152
**Body temperature, °C**	36.9 ± 0.6	37.0 ± 0.5	36.9 ± 0.6	<0.001
**Combined comorbidity, n (%)**				
** Hypertension**	1,201 (21.4)	695 (21.0)	506 (21.8)	0.455
** Diabetes mellitus**	691 (12.3)	366 (11.1)	325 (14.0)	<0.001
** Cardiovascular disease**	224 (4.0)	121 (3.7)	103 (4.5)	0.135
** Bronchial asthma**	128 (2.3)	82 (2.5)	46 (2.0)	0.222
** COPD**	40 (0.7)	15 (0.5)	25 (1.1)	0.006
** Chronic kidney disease**	55 (1.0)	29 (0.9)	26 (1.1)	0.357
** Malignancy**	145 (2.6)	96 (2.9)	49 (2.1)	0.066
** Chronic liver disease**	83 (1.6)	35 (1.1)	48 (2.2)	0.002
** Autoimmune disease**	38 (0.7)	26 (0.8)	12 (0.5)	0.217
** Dementia**	224 (4.2)	153 (4.9)	71 (3.2)	0.002
**Accompanying symptom, n (%)**				
** Fever**	1,305 (23.2)	784 (23.7)	521 (22.5)	0.286
** Cough**	2,341 (41.6)	1,423 (43.0)	918 (39.6)	0.011
** Sputum**	1,619 (28.8)	1,027 (31.1)	592 (25.6)	<0.001
** Sore throat**	881 (15.7)	591 (17.9)	290 (12.5)	<0.001
** Rhinorrhea**	621 (11.0)	382 (11.6)	239 (10.3)	0.145
** Myalgia**	926 (16.5)	600 (18.1)	326 (14.1)	<0.001
** Fatigue**	234 (4.2)	134 (4.1)	100 (4.3)	0.626
** Dyspnea**	666 (11.8)	403 (12.2)	263 (11.4)	0.340
** Headache**	967 (17.2)	668 (20.2)	299 (12.9)	<0.001
** Altered mental status**	35 (0.6)	19 (0.6)	16 (0.7)	0.586
** Nausea/Vomiting**	244 (4.3)	176 (5.3)	68 (2.9)	<0.001
** Diarrhea**	518 (9.2)	317 (9.6)	201 (8.7)	0.245
**Laboratory data**				
** WBC, μL**	6,126 ± 2,824	6,051 ± 2,681	6,243 ± 3,033	0.035
** Lymphocyte, %**	29.1 ± 11.7	30.1 ± 11.1	27.6 ± 12.3	<0.001
** Platelet, x10** ^**3**^ **/μL**	236.7 ± 82.9	244.6 ± 81.4	224.3 ± 83.6	<0.001
** Hemoglobin, g/dL**	13.3 ± 1.8	12.7 ± 1.4	14.2 ± 1.9	<0.001
** Hematocrit, %**	39.2 ± 5.0	37.8 ± 4.2	41.5 ± 5.3	<0.001
**Disease severity**				<0.001
** No limit of activity**	4,455 (79.5)	2,648 (80.5)	1,807 (78.2)	0.041
** Limit of activity but no O**_**2**_	330 (5.9)	212 (6.4)	118 (5.1)	0.037
** O**_**2**_ **with nasal prong**	469 (8.4)	268 (8.1)	201 (8.7)	0.458
** O**_**2**_ **with facial mask**	43 (0.8)	20 (0.6)	23 (1.0)	0.102
** Non-invasive ventilation**	33 (0.6)	16 (0.6)	17 (0.7)	0.229
** Invasive ventilation**	19 (0.3)	9 (0.3)	10 (0.4)	0.312
** Multi-organ failure/ECMO**	11 (0.2)	4 (0.1)	7 (0.3)	0.131
** Death**	241 (4.3)	114 (3.5)	127 (5.5)	<0.001
**Admission site**				<0.001
** Intensive care unit**	189 (3.4)	74 (2.2)	115 (5.0)	
** General ward**	5,410 (96.6)	3,216 (97.8)	2,194 (95.0)	
**Relief of isolation, n (%)**	5,387 (95.7)	3,194 (96.5)	2,193 (94.5)	<0.001
**Duration of isolation, days**	24.0 (18.0–32.0)	24.0 (18.0–32.0)	24.0(18.0–31.0)	0.244

Data are mean ± SD or number (percentage) or median (quartile 1–3). BMI = body mass index; SBP = systolic blood pressure; DBP = diastolic blood pressure; COPD = chronic obstructive pulmonary disease; WBC = whole blood count; ECMO = extracorporeal membrane oxygenation.

Body temperature at initial diagnosis (37.0 ± 0.5°C vs. 36.9 ± 0.6°C, p <0.001) was higher in women. Among accompanying symptoms, the prevalence of cough (43.0% vs. 39.6%, p = 0.011), sputum (31.1% vs. 25 6%, p <0.001), sore throat (17.9% vs. 12.5%, p <0.001), myalgia (18.1% vs. 14.1%, p <0.001), headache (20.2% vs. 12.9%, p <0.001), and nausea/vomiting (5.3% vs. 2.9%, p <0.001) were higher in women compared with men. The white blood cell (WBC) count (6,051 ± 2,681 μL vs. 6,243 ± 3,033 μL, p = 0.035), hemoglobin (12.7 ± 1.4 g/dL vs. 14.2 ± 1.9 g/dL, p <0.001), and hematocrit (37.8 ± 4.2% vs. 41.5 ± 5.3%, p <0.001) were lower in women. Lymphocyte (30.1 ± 11.1% vs. 27.6 ± 12.3%, p <0.001) and platelet (244,638 ± 81,462 μL vs. 224,320 ± 83,692 μL, p <0.001) counts were higher in women compared with men. The median admission duration was 24 days (Interquertile range: 18 to 32 days).

**Fig 2:**
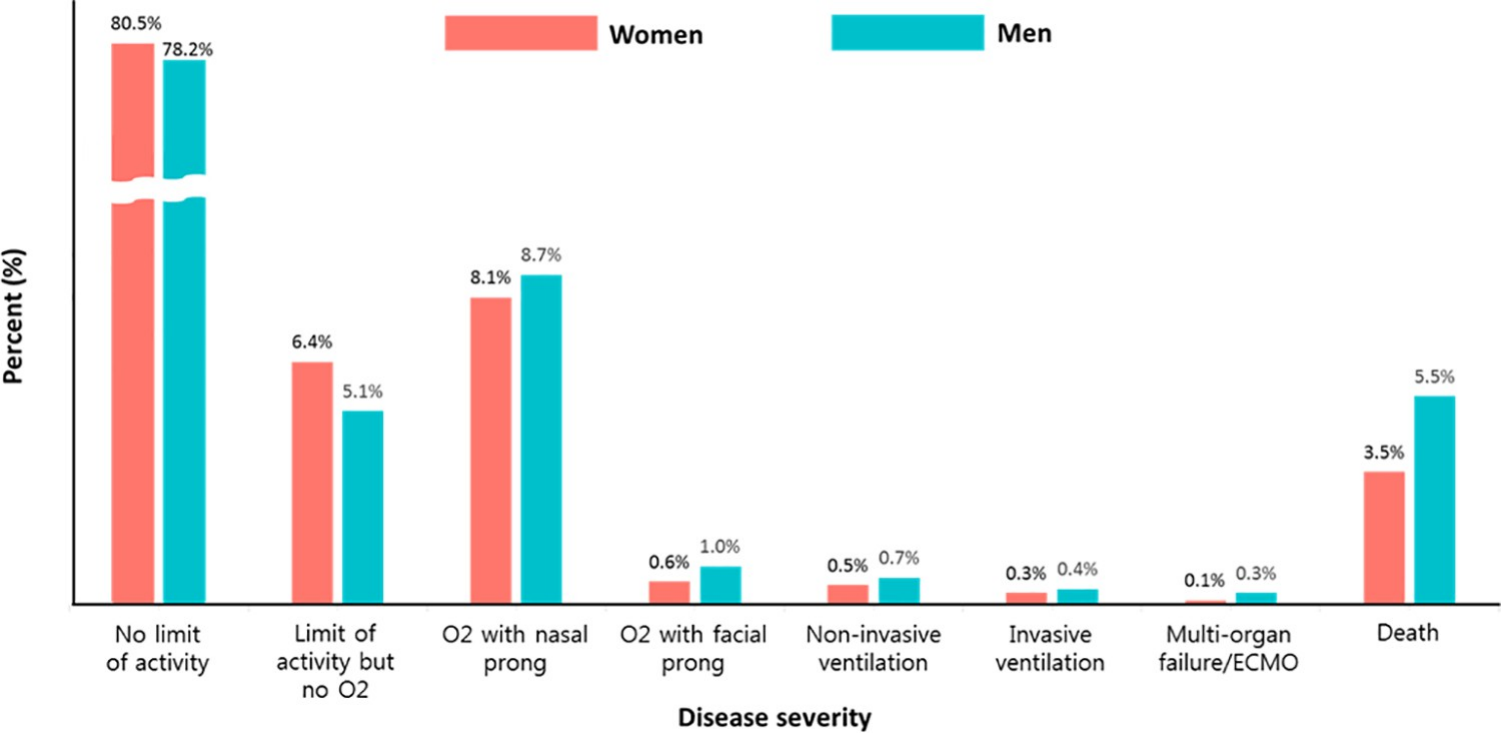
Sex-specific differences of in-hospital outcomes between women and men in COVID-19. ECMO = extracorporeal membrane oxygenation.

**Fig 3:**
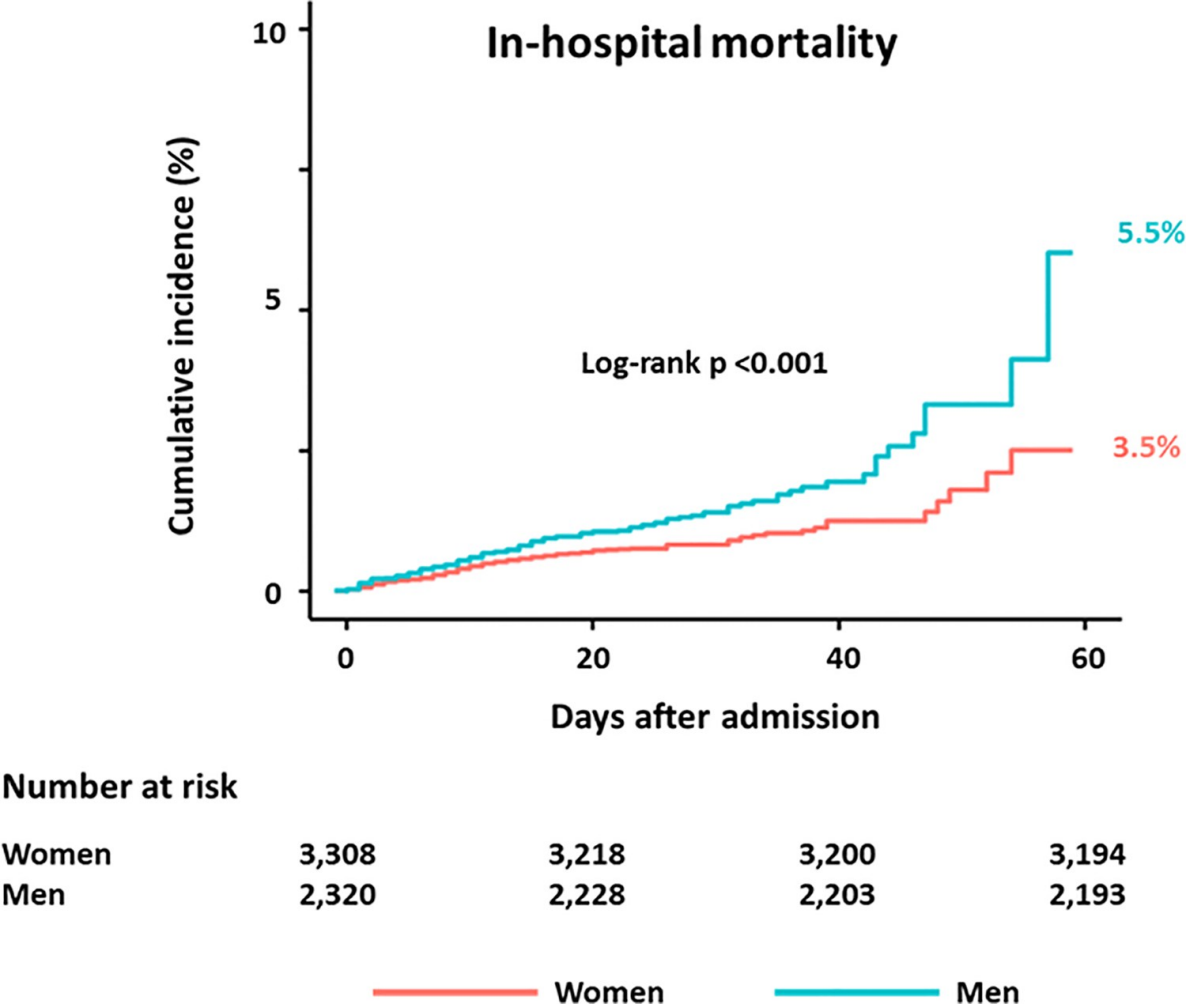
The Kaplan-Meier curves of cumulative incidences of in-hospital death between women and men.

The prevalence of COVID-19 infection and in-hospital mortality according to sex and age is presented in Fig 1. Above 40 years of age the prevalence of the disease was more common in women compared with men, and whilst in-hospital mortality increased with age for both sexes, it was always higher in men compared with the same aged women over 40. A comparison of in-hospital outcomes between women and men is presented in Fig 2. The cumulative incidences of in-hospital death were higher in men. The risk of death was significantly lower in women than in men (3.5% vs. 5.5%; hazard ratio (HR): 0.61; 95% CI: 0.47 to 0.79; p <0.001, log-rank p <0.001) (Fig 3). Sensitivity analyses using multivariable Cox regression (HR: 0.59; 95% CI: 0.41 to 0.85, p = 0.023) and propensity score matching (HR: 0.51; 95% CI: 0.30 to 0.86), p = 0.012) showed consistently lower risk of death in women compared with men (Table 2). In Table 2, there were no significant differences of clinical outcomes between women and men except in-hospital death.

**Table 2:** Comparison of clinical outcomes between women and men.

	Women	Men	Unadjusted		Multivariable-adjusted		Propensity score matched	
	(n = 3,308)	(n = 2,320)	HR (95% CI)	p-value	HR (95% CI)	p-value	HR (95% CI)	p-value
**No limit of activity**	2,648 (80.5)	1.807 (78.2)	0.99 (0.93–1.05)	0.779	1.10 (0.99–1.22)	0.077	1.09 (0.96–1.24)	0.180
**Limit of activity but no O**_**2**_	212 (6.4)	118 (5.1)	1.20 (0.96–1.50)	0.118	1.10 (0.74–1.64)	0.625	1.57 (0.95–2.61)	0.079
**O**_**2**_ **with nasal prong**	268 (8.1)	201 (8.7)	0.87 (0.72–1.04)	0.130	0.87 (0.67–1.13)	0.306	0.98 (0.72–1.34)	0.895
**O**_**2**_ **with facial mask**	20 (0.6)	23 (1.0)	0.55 (0.30–1.01)	0.053	0.73 (0.30–1.81)	0.497	0.46 (0.12–1.77)	0.259
**Non-invasive ventilation**	16 (0.6)	17 (0.7)	0.70 (0.34–1.41)	0.317	1.66 (0.60–4.60)	0.334	1.73 (0.41–7.25)	0.453
**Invasive ventilation**	9 (0.3)	10 (0.4)	0.80 (0.30–2.15)	0.655	0.38 (0.08–1.80)	0.224	0.15 (0.02–1.25)	0.079
**Multi-organ failure/ECMO**	4 (0.1)	7 (0.3)	0.27 (0.07–1.03)	0.056	Inf (0 .00-Inf)	0.841	2.14 (0.19–23.64)	0.534
**Death**	114 (3.5)	127 (5.5)	0.61 (0.47–0.79)	<0.001	0.59 (0.41–0.85)	0.023	0.51 (0.30–0.86)	0.012

Data are number (percentage). CI = confidence interval; HR = hazard ratio; ECMO = extracorporeal membrane oxygenation.

**Fig 4:**
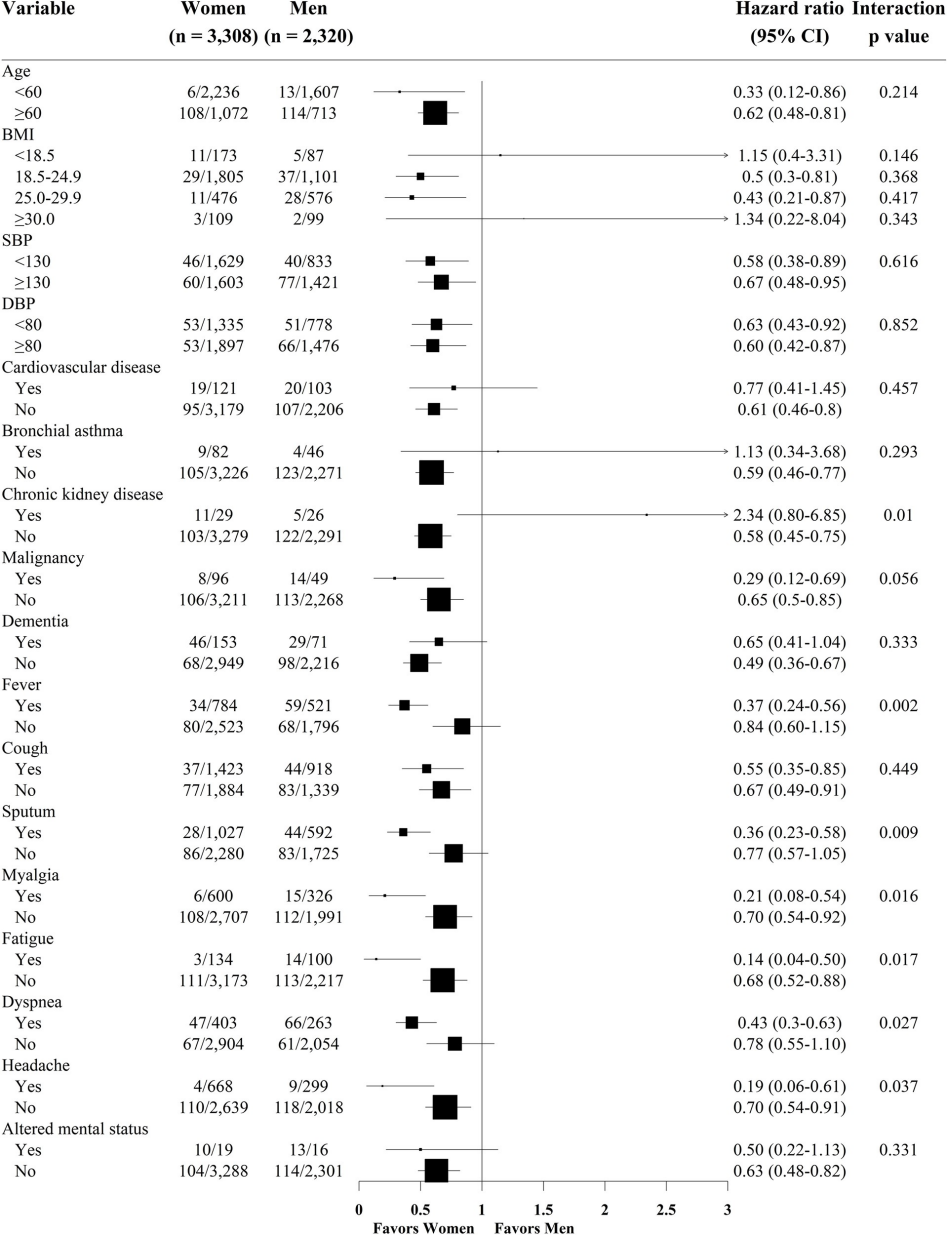
Subgroup analysis for sex-specific differences of in-hospital death by binary regression hazard ratio analysis in crude population. The results of exploratory subgroup analysis should be interpreted in the context of a significant interaction p value, and not the individual comparison in each subgroup, due to multiple testing issues. CI = confidence interval; BMI = body mass index; SBP = systolic blood pressure; DBP = diastolic blood pressure.

**Fig 5:**
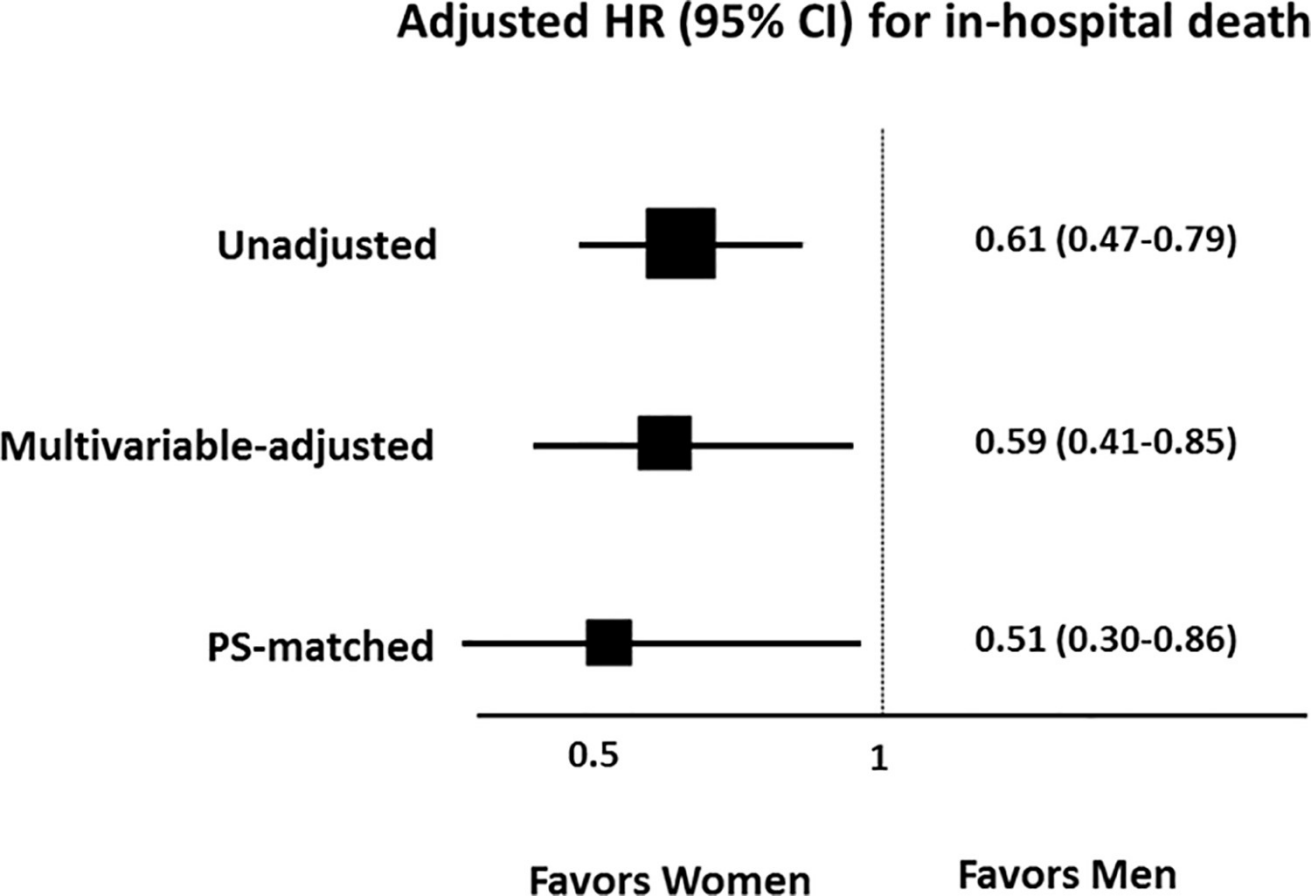
Exploratory subgroup analysis for in-hospital death. Women showed a significantly lower risk of in-hospital death than men, which was consistently observed after adjustment of baseline differences. In the multivariable Cox proportional hazard model, women were independently associated with a decreased risk of in-hospital death. PS = propensity score.

Multivariable Cox proportional hazard models identified independent predictors of the primary end point (Table 3). In women, age, body mass index (BMI), cardiovascular disease, chronic kidney disease, malignancy, dyspnea, lymphocyte count, and platelet count were independent predictors of in-hospital death. In men, age, heart rate, malignancy, autoimmune disease, dementia, fever, sputum, dyspnea, headache, and lymphocyte were independent predictors of in-hospital death. Fig 4 presents the prognostic impact of women among the various subgroups. The significantly lower risk of death in women than in men was consistent across all subgroups except some subgrouops with significant interaction p values. In Fig 5, women showed a significantly lower risk of in-hospital death than men, which was consistently observed after thorough sensitivity analyses for adjustment of baseline differences.

**Table 3:** Predictors of in-hospital mortality by logistic regression analysis in women and men.

Variable		Women			Men	
	HR	95% CI	p-value	HR	95% CI	p-value
**Age, per 10 years**	2.64	1.77–7.71	0.006	2.08	1.50–2.90	<0.001
**BMI, per categories**	0.83	0.58–1.18	<0.001	1.28	0.93–1.76	0.132
**Heart rate**	1.02	1.00–1.04	0.080	1.02	1.00–1.03	0.039
**Cardiovascular disease**	3.31	1.42–7.71	0.006	0.51	0.22–1.21	0.126
**Bronchial asthma**	2.88	0.90–9.22	0.075	0.49	0.09–2.79	0.422
**Chronic kidney disease**	3.98	1.32–12.0	0.014	0.63	0.12–3.34	0.587
**Malignancy**	3.46	1.06–11.3	0.040	2.79	1.14–6.83	0.025
**Autoimmune disease**	0	0-Inf	0.996	16.16	1.84–141.80	0.012
**Dementia**	1.97	0.89–4.36	0.942	4.57	1.98–10.57	<0.001
**Fever**	0.84	0.34–3.11	0.714	4.18	1.96–8.89	<0.001
**Sputum**	1.18	0.53–2.64	0.681	2.50	1.17–5.34	0.018
**Dyspnea**	4.28	2.05–8.91	<0.001	2.12	1.12–4.02	0.022
**Headache**	0.29	0.07–1.24	0.094	0.20	0.04–0.90	0.036
**Altered mental status**	1.24	0.18–8.32	0.827	3.29	0.93–11.55	0.638
**Lymphocyte, %**	0.93	0.89–0.96	<0.001	0.94	0.90–0.98	0.004
**Platelet, x10** ^**4**^ **/μL**	0.95	0.91–1.00	0.035	0.97	0.93–1.00	0.087
**Hematocrit, %**	0.93	0.80–1.09	0.388	0.92	0.83–1.02	0.841

HR = hazard ratio; CI = confidence interval; BMI = body mass index, less than 18.5 as 1, 18.5 to 22.9 as 2, 23.0 to 24.9 as 3, 25.0 to 29.9 as 4, more than or equal to 30 as 5.

## Discussion

The main findings of this study were as follows. First, women had a significantly lower risk of in-hospital death compared to men, which was maintained even after adjustment of baseline differences. Second, in the multivariable Cox proportional hazard model, women were independently associated with a decreased risk of in-hospital death. Third, the significantly lower risk of in-hospital death in women compared with men was consistently observed in various subgroups.

The first reports of COVID-19 suggested a sex imbalance with regards to detected cases and case fatality rate, with several subsequent studies suggesting that more men develop serious symptoms and have a higher mortality compared with women, potentially due to sex-based immunological or gendered differences [7–9]. However, sex-based disaggregated data of mortality from COVID-19 are still not available from all countries and a thorough analysis of sex-specific differences of mortality is currently lacking [4, 10].

Recently the *Global Health 50/50 research initiative* presented an interesting overview of sex-based disaggregated data from countries worldwide, clearly demonstrating similar numbers of cases among men and women, but with an increased case fatality rate in men (https://globalhealth5050.0rg/covid19/) [11]. Previous Korean national data during the initial period of COVID-19 infection showed that the case fatality rate and mortality were higher in men than women [12]. Similarly, our study showed that the cumulative incidence of in-hospital mortality from COVID-19 was higher in men compared with women (3.5% vs. 5.5%, p <0.001); our data show that 60% more hospitalized men died from COVID-19 than women. The higher mortality for men from COVID-19 does not directly imply that they are more vulnerable to the disease than women. For example, men had a higher prevalence of DM, cardiovascular disease, COPD, and chronic liver disease compared with women in this study, and these sex-specific differences could be contributing factors for the sex-biased mortality from COVID-19. Our study, however, confirms the robustness of these sex-specific differences with the consistently lower rates of in-hospital mortality from COVID-19 in women, observed even after multivariable adjustment and propensity score matching analysis (3.5% in women vs. 6.8% in men, p = 0.010). These significant differences in the men to women COVID-19 case fatality ratio can be observed between European and Asian countries, and these case fatality rates are relatively homogenous and range between 1.7–1.8 in men compared to women [11, 13–15]. Previous report including 38 countries provided sex-disaggregated data for a men bias in COVID-19 mortality such as higher 1.7 times of men case mortality ratio (women mortality 4.4 (95% CI 3.4–5.5) vs. men mortality 7.3 (95% CI 5.4–9.2) [13], which is consistent with our result of higher 1.6 times of men mortality than women (S1 Fig). This means that a consistent biological phenomenon may be operating, independent of country-specific demographics and national strategies.

Although the mechanisms and pathogenesis underlying these sex-specific differences are not fully understood, several studies suggested that the difference in immune system function between women and men could be an important determinant. Women are known to show a robust immune response to pathogens which could help them to better regulate viral load and viral clearance compared with men [16]. Since many immune genes are present on the X chromosome, the XX and XY genetic constitutions may contribute to COVID-19 severity and mortality [17]. Other differences including steroid hormone and sex organs such as testis and ovaries could also play an important role in pathogenesis. Estrogen in women can have immune-enhancing effects, while testosterone can have immune-suppressive effects [18–20]. In addition, several clinical trials highlight the relevance of sex differences in the renin angiotensin aldosterone system (RAAS), and there is increasing evidence that sex and sex hormones affect many components of circulating as well as tissue-based RAAS, including angiotensin converting enzyme (ACE) 2 and cellular serine protease TMPRSS2 [11, 21, 22]. The latter has been suggested to account for the higher mortality seen in men affected by COVID-19, and is of particular interest in the treatment of COVID-19, as a protein that primes severe acute respiratory syndrome coronavirus 2 (SARS-CoV-2) entry into cells [23, 24].

Lastly, when considering sex-specific differences in mortality, we should also consider how sex interacts with gender to influence vulnerability. Gender differences include differences in social and economic consequences as a result of the pandemic, including risk of domestic violence, economic and job insecurity, and increased domestic workload [4, 11, 25]. Contrary to other reports on COVID-19, we showed that the number of women infected with COVID-19 was 1.4 fold higher than men (59% vs. 41%), despite a women to men ratio of 50% in the general population. In South Korea, specific religious groups were associated with the largest number of COVID-19 infected patients [26], and the predominance of women in these religious groups could have influenced the female bias in Korean COVID-19 outbreak. Additionally, the occupational hazards of a crowded workplace, such as a call center (affected patients were predominantly women) could be a risk factor for COVID-19 infection in South Korea [27]. Finally, womens role as caregivers within the health system and at home may place them at increased risk of infection, and women are more likely to care for children or other family members who are ill [4]. Therefore, these social and cultural factors could have led to sex-specific differences in COVID-19, and more research is needed to understand how sex and gender are causing differential clinical outcomes and effects related to COVID-19 between women and men.

There are some limitations to our study that need consideration. First, this study has an innate limitation regarding its observational nature with registry data. For instance, men were more needed oxygen with activity and more went straight to the ICU compared to women, and these baseline differences could significantly affect outcomes. However, with the extensive sensitivity analyses, the confounders were adjusted to minimize the bias from different baseline characteristics. Second, this study used the national database of KCDC, in which the individual detailed characteristics relevant to sex and gender including socioeconomic status, smoking history, immunological condition, and unreported co-morbid conditions were not recorded. Third, we performed comparative analyses using surveillance data, which could not show information on precise clinical management, medicine, and sufficient laboratory and imaging data such as markers of disease severity at admission and chest computed tomogrpahy images. Finally, our results are not applicable to all countries and cases of COVID-19 owing to our analysis being confined to South Korea.

In conclusion, women had a significantly lower risk of in-hospital death amongst those patients hospitalized with COVID-19 infection in South Korea.

## Supporting information

S1 Fig Men predominance in COVID-19 mortality (deaths divided by confirmed cases) in worldwide and South Korea. A men to women mortality ratio of 1 reflects sex balance, the blue bars reflect men predominance. The worldwide data were obtained from (12) Scully EP et al. Nat Rev Immunol. 2020;20(7):442–7.
(TIF)

S1 File (DOCX)
