# Supplementary figures and images for: Sex-specific difference of in-hospital mortality from COVID-19 in South Korea
Source: PLoS One. 2022 Jan 24;17(1):e0262861. doi: 10.1371/journal.pone.0262861 (PMC8786158; doi:10.1371/journal.pone.0262861)

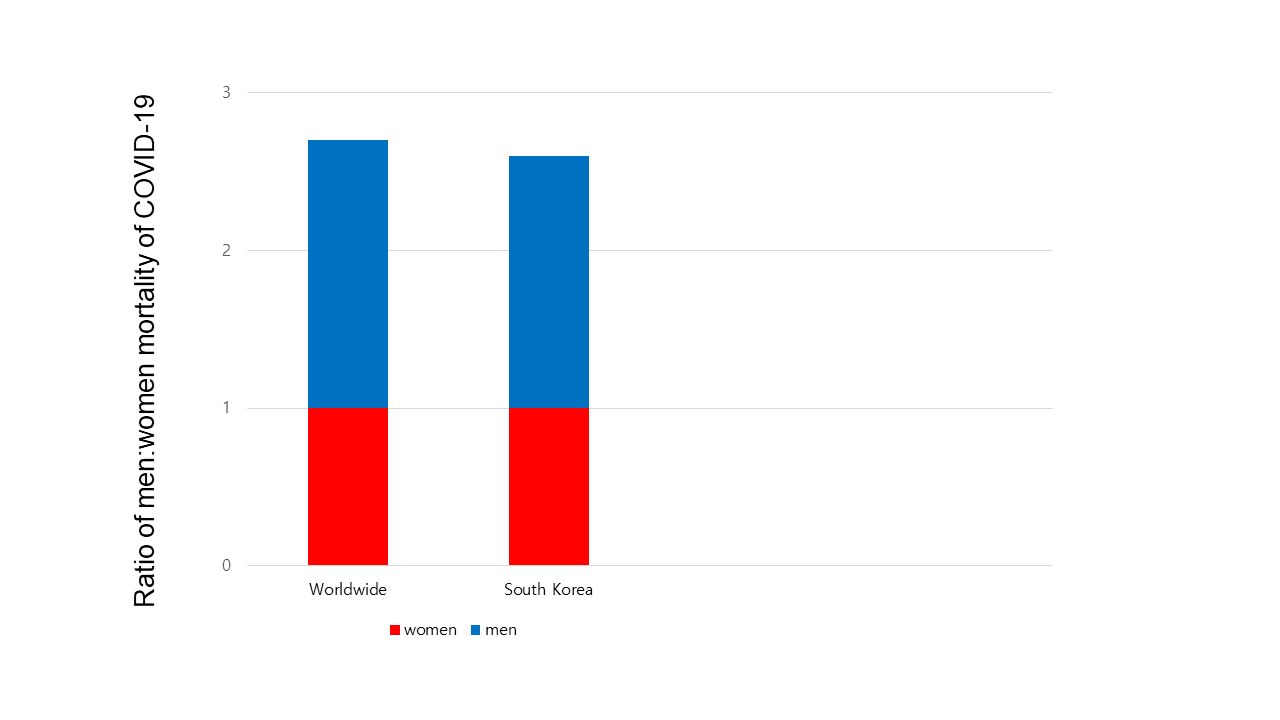

Supplement: S1 Fig — A men to women mortality ratio of 1 reflects sex balance, the blue bars reflect men predominance. The worldwide data were obtained from (12) Scully EP et al. Nat Rev Immunol. 2020;20(7):442–7. (TIF) [file pone.0262861.s001.tif]
