# Supplementary material for: Sex-specific difference of in-hospital mortality from COVID-19 in South Korea
Source: PLoS One. 2022 Jan 24;17(1):e0262861. doi: 10.1371/journal.pone.0262861 (PMC8786158; doi:10.1371/journal.pone.0262861)
Supplement: S1 File — (DOCX) [file pone.0262861.s002.docx]

**Supplementary Methods**

**Statistical analysis**

Categorical variables were presented as numbers and relative frequencies (percentages) and were compared using the Chi-squared test. Continuous variables were expressed as mean ± standard deviation or median (Q1-Q3), according to whether they were normally distributed or not, and were compared using the independent sample t test or Mann-Whitney test, as appropriate. Covariates included in multivariable model were selected if they were significantly different between the 2 groups or had predictive values, which are listed as follows: age, body mass index (BMI), systolic blood pressure (SBP), diastolic blood pressure (DBP), heart rate, body temperature, fever, cough, sputum, sore throat, rhinorrhea, myalgia, fatigue, dyspnea, headache, altered mental status, nausea/vomiting, diarrhea, diabetes mellitus (DM), hypertension (HTN), cardiovascular disease, bronchial asthma, chronic obstructive pulmonary disease (COPD), chronic kidney disease, malignancy, chronic liver disease, autoimmune disease, dementia, hemoglobin, hematocrit, lymphocyte, platelet, whole blood count (WBC). A multivariable logistic regression model was used to generate propensity-scores. All the available covariates were included to this model, precisely following the recommendations of analysis using propensity-score [1]. For the propensity-score matching, a 1:1 matching process without replacements was performed with a caliper width of 0.01, yielding 621 patients in the women group matched with 621 controls in the men group. Balance between the 2 groups after propensity-score matching was assessed by calculating standardized mean differences of covariates used in propensity-score generation. Standardized mean differences after propensity-score matching were within 0.1 across all matched covariates, demonstrating successful balance achievement between comparative groups (Supplementary Table 1). The hazard ratios (HR) and 95% credible intervals were calculated as a result of Cox regression. The credible intervals of the hazard ratios (HR), which did not include 1, were considered significant. To identify independent predictors of in-hospital death, we used multivariable Cox proportional hazard model. C-statistics with 95% confidence intervals (CI) were calculated to validate the discriminant function of the model. In addition, comparisons of the primary outcome between women and men groups according to the exploratory subgroups of interest were followed, and the interaction between in-hospital death and these covariates was assessed with Cox regression model. In all the analysis, the participating centers were included as random effects. All probability values were two-sided and p values <0.05 were considered statistically significant. The Statistical analyses were performed using R version 4.0.2 (R Foundation for Statistical Computing, Vienna, Austria).

**Supplementary Table 1.** Baseline clinical characteristics and outcomes by Propensity-score matched analysis, and standardized mean differences of variables among unadjusted and propensity-score matched.

| Variables | Total  (n = 1,242) | Women  (n = 621) | Men  (n = 621) | p-value | Standardized mean differences | |
| --- | --- | --- | --- | --- | --- | --- |
|  |  |  |  |  | Unadjusted (n = 5,628) | PS matched (n = 1,242) |
| Age, years |  |  |  |  | 0.284 | 0.076 |
| 0 - 9 | 18 (1.4) | 9 (1.4) | 9 (1.4) | 1.000 | 0.065 | <0.001 |
| 10 - 19 | 40 (3.2) | 22 (3.5) | 18 (2.9) | 0.520 | 0.089 | 0.036 |
| 20 – 29 | 195 (15.7) | 99 (15.9) | 96 (15.5) | 0.815 | 0.162 | 0.013 |
| 30 - 39 | 109 (8.8) | 53 (8.5) | 56 (9.0) | 0.764 | 0.088 | 0.017 |
| 40 - 49 | 136 (11.0) | 71 (11.4) | 65 (10.5) | 0.586 | 0.141 | 0.031 |
| 50 - 59 | 252 (20.3) | 119 (19.2) | 133 (21.4) | 0.323 | 0.131 | 0.056 |
| 60 - 69 | 227 (18.3) | 112 (18.0) | 115 (18.5) | 0.826 | 0.001 | 0.013 |
| 70 - 79 | 169 (13.6) | 87 (14.0) | 82 (13.2) | 0.679 | 0.018 | 0.023 |
| ≥80 | 96 (7.7) | 49 (7.9) | 47 (7.6) | 0.832 | 0.095 | 0.012 |
| BMI, kg/m^2^ |  |  |  |  | 0.384 | 0.059 |
| <18.5 (Underweight) | 78 (6.3) | 40 (6.4) | 38 (6.1) | 0.815 | 0.090 | 0.013 |
| 18.5 – 24.9 (Normal) | 789 (63.5) | 389 (62.6) | 400 (64.4) | 0.517 | 0.239 | 0.037 |
| 25.0 – 29.9 (Overweight) | 322 (25.9) | 167 (26.9) | 155 (25.0) | 0.437 | 0.289 | 0.044 |
| ≥30.0 (Obesity) | 53 (4.3) | 25 (4.0) | 28 (4.5) | 0.674 | 0.050 | 0.024 |
| SBP, mmHg |  |  |  |  | 0.325 | 0.037 |
| <130 | 538 (43.3) | 271 (43.6) | 267 (43.0) | 0.819 | 0.274 | 0.013 |
| ≥130 | 704 (56.7) | 350 (56.4) | 354 (57.0) | 0.819 | 0.274 | 0.013 |
| DBP, mmHg |  |  |  | 0.932 | 0.184 | 0.038 |
| <80 | 463 (37.3) | 230 (37.0) | 233 (37.5) | 0.860 | 0.140 | 0.010 |
| ≥80 | 779 (62.7) | 391 (63.0) | 388 (62.0) | 0.860 | 0.140 | 0.010 |
| Heart rate, beats/min | 86 ± 16 | 86 ± 16 | 85 ± 16 | 0.587 | 0.039 | 0.031 |
| Body temperature, ℃ | 36.9 ± 0.6 | 36.9 ± 0.6 | 36.9 ± 0.6 | 0.754 | 0.143 | 0.018 |
| Combined comorbidity, n (%) |  |  |  |  |  |  |
| Hypertension | 348 (28.0) | 179 (28.8) | 169 (27.2) | 0.527 | 0.020 | 0.036 |
| Diabetes mellitus | 220 (17.7) | 109 (17.6) | 111 (17.9) | 0.882 | 0.090 | 0.008 |
| Cardiovascular disease | 76 (6.1) | 39 (6.3) | 37 (6.0) | 0.813 | 0.040 | 0.013 |
| Bronchial asthma | 30 (2.4) | 13 (2.1) | 17 (2.7) | 0.460 | 0.033 | 0.042 |
| COPD | 12 (1.0) | 6 (1.0) | 6 (1.0) | 1.000 | 0.072 | <0.001 |
| Chronic kidney disease | 19 (1.5) | 9 (1.4) | 10 (1.6) | 0.817 | 0.025 | 0.013 |
| Malignancy | 45 (3.6) | 21 (3.4) | 24 (3.9) | 0.649 | 0.050 | 0.026 |
| Chronic liver disease | 24 (1.9) | 12 (1.9) | 12 (1.9) | 1.000 | 0.083 | <0.001 |
| Autoimmune disease | 8 (0.6) | 4 (0.6) | 4 (0.6) | 1.000 | 0.035 | <0.001 |
| Dementia | 53 (4.3) | 27 (4.3) | 26 (4.2) | 0.888 | 0.086 | 0.008 |
| Accompanying symptom, n (%) |  |  |  |  |  |  |
| Fever | 283 (22.8) | 136 (21.9) | 147 (23.7) | 0.457 | 0.029 | 0.042 |
| Cough | 532 (42.8) | 267 (43.0) | 265 (42.7) | 0.909 | 0.069 | 0.007 |
| Sputum | 348 (28.0) | 173 (27.9) | 175 (28.2) | 0.899 | 0.122 | 0.007 |
| Sore throat | 172 (13.8) | 87 (14.0) | 85 (13.7) | 0.869 | 0.150 | 0.009 |
| Rhinorrhea | 125 (10.1) | 64 (10.3) | 61 (9.8) | 0.777 | 0.040 | 0.016 |
| Myalgia | 188 (15.1) | 95 (15.3) | 93 (15.0) | 0.874 | 0.111 | 0.009 |
| Fatigue | 65 (5.2) | 32 (5.2) | 33 (5.3) | 0.899 | 0.013 | 0.007 |
| Dyspnea | 178 (14.3) | 86 (13.8) | 92 (14.8) | 0.627 | 0.026 | 0.028 |
| Headache | 178 (14.3) | 84 (13.5) | 94 (15.1) | 0.418 | 0.197 | 0.046 |
| Altered mental status | 5 (0.4) | 2 (0.3) | 3 (0.5) | 0.654 | 0.015 | 0.025 |
| Nausea/Vomiting | 65 (5.2) | 34 (5.5) | 31 (5.0) | 0.702 | 0.120 | 0.022 |
| Diarrhea | 89 (7.2) | 41 (6.6) | 48 (7.7) | 0.441 | 0.032 | 0.044 |
| Laboratory data |  |  |  |  |  |  |
| WBC, µL | 6,169 ± 2,867 | 6,130 ± 2,589 | 6,209 ± 3,122 | 0.627 | 0.067 | 0.028 |
| Lymphocyte, % | 27.8 ± 11.6 | 27.5 ± 10.9 | 28.1 ± 12.3 | 0.324 | 0.215 | 0.056 |
| Platelet, x10^3^/µL | 236.2 ± 84.0 | 237.5 ± 79.3 | 234.8 ± 88.5 | 0.574 | 0.246 | 0.032 |
| Hemoglobin, g/dL | 13.5 ± 1.6 | 13.4 ± 1.3 | 13.5 ± 1.9 | 0.784 | 0.888 | 0.016 |
| Hematocrit, % | 39.6 ± 4.6 | 39.6 ± 4.0 | 39.6 ± 5.1 | 0.975 | 0.779 | 0.002 |
| Disease severity |  |  |  |  | 0.136 | 0.230 |
| No limit of activity | 926 (74.6) | 471 (75.8) | 455 (73.3) | 0.297 | 0.055 | 0.059 |
| Limit of activity but no O_2_ | 63(5.1) | 38 (6.1) | 25 (4.0) | 0.093 | 0.057 | 0.096 |
| O_2_ with nasal prong | 160 (12.9) | 79 (12.7) | 81 (13.0) | 0.865 | 0.020 | 0.010 |
| O_2_ with facial mask | 10 (0.8) | 3 (0.5) | 7 (1.1) | 0.204 | 0.044 | 0.072 |
| Non-invasive ventilation | 8 (0.6) | 5 (0.8) | 3 (0.5) | 0.478 | 0.032 | 0.040 |
| Invasive ventilation | 8 (0.6) | 1 (0.2) | 7 (1.1) | 0.033 | 0.027 | 0.121 |
| Multi-organ failure/ECMO | 3 (0.2) | 2 (0.3) | 1 (0.2) | 0.563 | 0.039 | 0.033 |
| Death | 64 (5.2) | 22 (3.5) | 42 (6.8) | 0.010 | 0.098 | 0.146 |
| Admission site |  |  |  | 0.005 | 0.147 | 0.159 |
| Intensive care unit | 59 (4.8) | 19 (3.1) | 40 (6.4) |  |  |  |
| General ward | 1,183 (95.2) | 602 (96.9) | 581 (93.6) |  |  |  |
| Relief of isolation, n (%) | 1,178 (94.8) | 599 (96.5) | 579 (93.2) | 0.010 | 0.098 | 0.146 |
| Duration of isolation, days | 26.0 ± 11.4 | 25.5 ± 11.2 | 26.5 ± 11.6 | 0.098 | 0.032 | 0.094 |

Data are mean ± SD or number (percentage). PS: propensity score; BMI: body mass index; SBP: systolic blood pressure; DBP: diastolic blood pressure; COPD: chronic obstructive pulmonary disease; WBC: whole blood count; ECMO: extracorporeal membrane oxygenation

**References**

1. Austin PC, Stuart EA. Moving towards best practice when using inverse probability of treatment weighting (IPTW) using the propensity score to estimate causal treatment effects in observational studies. Stat Med 2015;34(28):3661-3679.
